# The existential risk space of climate change

**DOI:** 10.1007/s10584-022-03430-y

**Published:** 2022-09-12

**Authors:** Christian Huggel, Laurens M. Bouwer, Sirkku Juhola, Reinhard Mechler, Veruska Muccione, Ben Orlove, Ivo Wallimann-Helmer

**Affiliations:** 1 Department of Geography, University of Zurich, Zurich, Switzerland; 2 Center of Competence for Sustainable Finance, University of Zurich, Zurich, Switzerland; 3 Climate Service Center Germany (GERICS), Helmholtz-Zentrum Hereon, Hamburg, Germany; 4 Ecosystems and Environment Research Programme, Helsinki Institute of Sustainability Science (HELSUS), University of Helsinki, Helsinki, Finland; 5 International Institute for Applied System Analysis (IIASA), Laxenburg, Austria; 6 School of International and Public Affairs, Columbia University, New York City, NY USA; 7 Climate School, Columbia University, New York City, NY USA; 8 Environmental Sciences and Humanities Institute, University of Fribourg, Fribourg, Switzerland

**Keywords:** Existential risks, Climate change, Physical survival, Basic human needs, Well-being, Habitability

## Abstract

Climate change is widely recognized as a major risk to societies and natural ecosystems but the high end of the risk, i.e., where risks become existential, is poorly framed, defined, and analyzed in the scientific literature. This gap is at odds with the fundamental relevance of existential risks for humanity, and it also limits the ability of scientific communities to engage with emerging debates and narratives about the existential dimension of climate change that have recently gained considerable traction. This paper intends to address this gap by scoping and defining existential risks related to climate change. We first review the context of existential risks and climate change, drawing on research in fields on global catastrophic risks, and on key risks and the so-called Reasons for Concern in the reports of the Intergovernmental Panel on Climate Change. We also consider how existential risks are framed in the civil society climate movement as well as what can be learned in this respect from the COVID-19 crisis. To better frame existential risks in the context of climate change, we propose to define them as those risks that threaten the existence of a subject, where this subject can be an individual person, a community, or nation state or humanity. The threat to their existence is defined by two levels of severity: conditions that threaten (1) survival and (2) basic human needs. A third level, well-being, is commonly not part of the space of existential risks. Our definition covers a range of different scales, which leads us into further defining six analytical dimensions: physical and social processes involved, systems affected, magnitude, spatial scale, timing, and probability of occurrence. In conclusion, we suggest that a clearer and more precise definition and framing of existential risks of climate change such as we offer here facilitates scientific analysis as well societal and political discourse and action.

## Introduction

Climate change has long been recognized as a major threat^1^ to societies and ecosystems. Research has provided a substantial body of evidence and extended the understanding of the impacts and risks of climate change, bringing these to the attention of policymakers, particularly through IPCC reports published from 1996 to 2022. For some species and ecosystems, such as coral reefs, possible extinction and destructive effects have been tied to specific levels of warming (IPCC 2018, 2019a, 2022). For human systems, however, specification of the type, magnitude, extent, and timing of such threats remains elusive. Some evidence has emerged on severe risks to human health (Im et al. 2017; Pörtner 2021; Ebi et al. 2021b) and to the viability of coastal communities (Mechler et al. 2020). Important gaps in understanding exist for the levels of vulnerability, adaptive capacity, and resilience that may define limits of adaptation and habitability (Horton et al. 2021; Thomas et al. 2021); there is also limited understanding on the severity of consequences for risks which propagate and cascade through human and coupled socio-environmental systems (Pescaroli and Alexander 2018; Renn et al. 2019; Lawrence et al. 2020; Simpson et al. 2021).

The narratives about the existential dimension of climate change for societies and for humanity as a whole have recently received considerable traction as it is one of the dominant themes articulated by climate movements across the globe. People who participate in these movements, such as the Extinction Rebellion and the Sunrise Movement, portray climate change as an existential threat to their future and to the future of humanity (Han and Ahn 2020). Even in mainstream politics, leaders have recently issued public statements that climate change is a matter of survival (Angela Merkel at the 2020 World Economic Forum), or the “existential crisis of our time” (Joe Biden at the April 2021 Leaders Summit on Climate). Government leaders of Small Island States have been using the same rhetoric for a much longer time. Although recent research and high-level science-policy reports have started to make reference to existential threats, a more substantiated engagement of a larger body of research on the nature of this existential threat has not yet taken place; in fact, there is very little research on the existential space of climate change as we call it further on. This gap sharply contrasts with the urgent need to provide necessary evidence for policy action and that is perceived in part of the general public (Wilson and Orlove 2021). At the same time, the issue is not only about the limited scientific understanding of existential risks in relation with climate change, but also about the discrepancy of the use of the term “existential” between the scientific community and the public. The public discourse over the past years suggests that “existential” is used for risks that science, and in particular the IPCC, considers as high or very high (Zommers et al. 2020).

This paper, hence, contributes to a better understanding of the existential risk space of climate change through scoping and an improved definition of what it refers to, and a discussion of key foundations to decide between existential and other levels of risk. We thereby understand the term “space” in terms of both analytical and geographic space. A better understanding and fuller evidence in this field could potentially contribute to better position the debates about climate change and existential threats in civil society and policy. In addition, it could improve scientific analysis of existential risks actions.

We first review the context of existential risk research, and its relation to and its representation in the context of climate change. We then identify key steps to provide a tangible definition of existential risks in relation to climate change and then we scope important dimensions of existential risks. We conclude by outlining some implications for risk analysis and management in research and policy.

## Definitions, concepts, and narratives of existential risks

Different definitions and scopes of existential risks, not only climate related, have been developed by researchers and civil society. We discuss four of these: global catastrophic risk, climate change risk and the Reasons for Concern (RFC) of the IPCC, civil society perceptions of risks emerging from and reflected by the climate movement, and definitions of risk stemming from the COVID-19 pandemic. While we consider these four narratives to be important and partly connected entry points for framing existential risks under climate change, we do not assume them to be a comprehensive or exhaustive list, nor are we able to do full justice to the depth and complexity of each of them. However, by discussing various narratives, we will be able to define existential risks with their elements and dimensions.

### Global catastrophic risk

The research community on existential risks typically defines existential risks as threats that could cause the extinction of humanity or destroy the potential of intelligent life on Earth (Bostrom 2002). Scholars distinguish between natural existential risks, such as a large asteroid impact on earth or a supervolcanic eruption, and anthropogenic existential risks, including those related to nuclear war, artificial intelligence, pandemics, and climate change (Bostrom 2013). Existential risks can be seen either as a subset or a synonym for global catastrophic risks (GCR), which are defined as those risks that threaten the entirety of human population and civilization (Baum and Barrett 2018). The common and distinguishing scope of these existential and global catastrophic risks is the focus on events or scenarios that place a large proportion or the entirety of humans at risk of death (Ó hÉigeartaigh 2017), although it is often not detailed over which periods of times such catastrophes would unfold. In this logic, more local catastrophes, and even major disasters like Chernobyl in 1986, the 2004 Indian Ocean Tsunami, or the Spanish influenza in 1918–1920, would not qualify as this kind of risk. Similarly, the COVID-19 pandemic, although with unprecedented global and probably long-lasting effects on people, society, and economy, would not qualify as an existential or global catastrophic risk because it is not considered a threat to the survival of humanity. Torres (2019) provides an analysis of five types of existential risks that encompass human extinction, civilizational collapse, permanent, drastic or significant losses of expected value or potential, and a pan-generational crushing catastrophe, which he compiles in a matrix of scope (from personal and local to pan-generational) and severity (from imperceptible to crushing).

Torres (2019) thereby strongly advocates for more analysis and efforts to communicate those risks to the public. In fact, the development of more refined frameworks may facilitate a more granular understanding of threats and affected systems, and thus an understanding of the type and class of risks. For example, Avin et al. (2018) distinguish three key components of GCR, i.e. a critical system whose safety boundaries are breached by a potential threat; the mechanisms of the spread of the threat over the entire globe; and the failure to prevent or mitigate such system failure and the spread of threat. At the scale of GCR, critical systems are defined as those that, if disturbed beyond a point on a certain scale, could substantially limit the ability of humanity to survive. The framework can, however, also be applied to risks with less drastic consequences than portrayed above (e.g., for COVID-19). In an influential report published more than 20 years ago, the German Advisory Council on Global Change developed a taxonomy of globally relevant risks where GCRs would fall into the highest risk space, the so-called prohibited area (WBGU 1998). Some scholars also underline the global nature pertaining to systemic risks (Renn et al. 2019) whose common characteristics include cascading impacts in complex and interconnected systems and the potential to induce breakdowns of entire sectors (e.g., healthcare, energy infrastructure, telecommunication, finance) (Kaufman and Scott 2003; Helbing 2013).

### Key risks and the “Reasons for Concern” of the IPCC

Existential risk is not a narrative or term that has been widely adopted or further developed by the climate change research community. Neither the concept of existential risks nor the term “existential” was used in the IPCC 5th Assessment Report (AR5), nor in the IPCC Special Reports of the 6th Assessment Cycle, i.e., the Special Report on Global Warming of 1.5°C (SR15) (IPCC 2018), on the Oceans and the Cryosphere (IPCC 2019a), and on Climate Change and Land (IPCC 2019b). An exception is a Cross-Chapter Box on residual risks, limits to adaptation and Loss and Damage (L&D) in the IPCC SR15, where reference is made to existential risks as a perspective on the Loss and Damage policy discourse (Roy et al. 2018). Stakeholder interviews on Loss and Damage showed that the existential perspective is prevalent in the UNFCCC among other perspectives, referring to climate change as unavoidable and having irreversible impacts for some communities and systems (Boyd et al. 2017). The IPCC SR15 furthermore presents evidence of relevance to the discussion on local existential risks in health systems (e.g., the proliferation of heatwaves in megacities) and in coastal systems, including in some small islands where compounding risks linked to sea-level rise and surge, salinization, heatwaves, and drought could lead to some degree of relocation or displacement during this century (Magnan et al. 2021). Such cases of impacts and risks might not be existential for humanity as a whole but certainly for those communities affected, especially in cases where loss of land, sovereign government, or cultural heritage cannot be accommodated by insurance schemes or other monetary mechanisms (Heyward 2014; Page and Heyward 2017; Wallimann-Helmer et al. 2019).

In the recently published IPCC AR6 report of Working Group II, broadly speaking, there are three narratives in which existential threat or risk is explicitly used (IPCC 2022): (i) sea-level rise posing an existential threat to Small Island States and low-lying coasts; (ii) Loss and Damage, in line with the analysis and discourse mentioned above for the IPCC SR15; (iii) relocation which can pose existential threats to sense of place, identity, and citizenship.

The IPCC AR5 introduced the concept of key risks that can potentially have severe adverse consequences for humans and socio-ecological systems (Oppenheimer et al. 2014). Criteria for identifying key risks include probability, timing, magnitude, systems affected and irreversibility of corresponding risks, and limitations to reduce risks through mitigation and adaptation (O’Neill et al. 2017). Notably, none of these key risks reaches a level where human civilization would be threatened, as it would be by GCR. The key risks were also intended to inform the Reasons for Concern (RFC), which are probably the risks treated in current and past IPCC reports that have the strongest resemblance to (*sensu* GCR).

The five RFCs are (Oppenheimer et al. 2014; O’Neill et al. 2022) risks to unique and threatened (eco-) systems (RFC1); risks from extreme climate events (RFC2); distribution of impacts (RFC3); aggregate (economic) impacts (RFC4); and risks from future large-scale discontinuities (RFC5) (Smith et al. 2001).

RFC1 and RFC5 encompass a number of disruptive processes in climate, cryosphere, and ecological systems with global-scale consequences, namely the collapse of the West Antarctic Ice Sheet (WAIS), the irreversible loss of the Greenland Ice Sheet, or the ecological transformation of the Amazon or boreal forest systems (Smith et al. 2009; O’Neill et al. 2017). The IPCC AR6 assessed the transition from low to moderate or high risks at lower temperature levels than previous IPCC Reports, e.g., RFC2 risks start to transition to a high level when global warming reached 1°C, or RFC5 transitions to moderate levels of risk already at 1°C warming, and to high risk levels between 1.5 and 2.5°C (O’Neill et al. 2022). RFC5 is underlaid by the concept of tipping points in climate and other natural and ecological systems which imply irreversible, large-scale processes with positive, self-reinforcing mechanisms if certain thresholds of warming are crossed. Global tipping cascades, involving for instance ice sheet collapse, Amazon rainforest transformation, or Arctic sea ice loss and permafrost thaw, are of particular concern and would pose an existential threat to civilization, as Lenton et al. (2019) suggest.

Consequences of such large-scale discontinuities as encompassed in RFC5 on human systems and civilization, however, are poorly analyzed in research and consequently are not fully assessed or treated in depth in the IPCC reports (Tol et al. 2006; Martens et al. 2010; Collins et al. 2019). Due to the large uncertainties about the exact physical processes of tipping point events, impacts on societies are difficult to assess. Collapse of WAIS and the Greenland Ice Sheet would result in about 10-m sea-level rise (Oppenheimer et al. 2019), making large areas of coastal regions and islands uninhabitable, though over a time scale of centuries if not millennia (Lenton et al. 2019). Such a scenario would represent an existential risk, i.e., a threat to the existence of hundreds of millions to billions of people. To what extent it would actually imply an imminent existential risk depends on the response where the long-time horizon could play a crucial aspect. However, the IPCC AR6 found limited evidence on the potential of adaptation to effectively reduce risks related to RFCs (O’Neill et al. 2022). In this context, it is pertinent to consider recent research that analyzes climate change–induced socio-economic tipping points where a socio-economic system through adaptation (or mitigation) transforms into a new, fundamentally different state (van Ginkel et al. 2020) which could potentially reduce existential risks to lower risk levels.

### Civil society climate movement

An additional narrative about the existential dimension of climate change has emerged, not from academic or policy contexts, but from the climate movement. In particular, the climate activists from “Fridays For Future” to “Extinction Rebellion” refer to the existential dimension, perceiving climate change as a threat to their future. The empirical research on this movement is still in its infancy (Fisher 2019; Knops 2021) but some survey-based studies suggest that participation of young people in climate strikes is related to risk perception (Brügger et al. 2020; Misch et al. 2021), in line with earlier studies (Wang et al. 2018). The youth movement has identified climate change as the greatest existential threat to the Earth and to human beings, and they consider this threat as a moral element of their narrative (Han and Ahn 2020). In an interview in 2019, Greta Thunberg stated that her message to the young people of the world is that we are facing right now an existential crisis, which she understood as the climate and ecological crisis (Thunberg 2019).

However, the thinking and theoretical fundaments underlying the narratives of the climate movement differ from the scientific understanding of climate risk. Recent empirical and theoretical research on the youth climate movement identified narratives of vernacular eschatology and postapocalyptics where temporality and imaginaries of collapse play an important role (Knops 2021; Friberg 2022). The term existential thus refers more to the need of a fundamental change which is also reflected by the constant critique of capitalism by the climate movement, and the request of drastic measures in line with changes in societal structures and economy, illustrated by the well-known slogan “system change not climate change” (SCNCC 2021).

At a first sight, the narratives of the climate movement may show some similarity with GCR but a deeper understanding of their fundaments as indicated above would not suggest so. Although the climate movement persistently urges politicians to listen to science (Svensson and Wahlström 2021), there is often no strong and explicit reference to scientific understanding of high risks of climate change. This may also be due to an insufficient framing and definition of the high risk and existential space of climate change from the side of science, hence underlying the importance of a clearer, more explicit and nuanced definition.

### Definitions and narratives from the COVID-19 crisis

The World Health Organization declared the COVID-19 outbreak a global pandemic in March 2020; scholars also referred to it as an “existential crisis” (Döring 2020; Ataguba and Ataguba 2020), an “existential risk” (Morens et al. 2020) or “existential threat” (Ebi et al. 2021a; Franke and Elliott 2021), or a “serious threat to humanity” (Gupta 2020). Pandemics are, in fact, recognized among the existential risks ( GCR), as outlined above (Ord 2020). However, many experts agree that COVID-19 does not pose an existential risk to human civilization, because the mortality of the disease is low among reproductive age groups at present and does not create a danger to the survival of humanity. Nevertheless, COVID-19 has been framed as an existential threat to people’s lives and livelihoods and to the well-functioning of societies, and used to justify or legitimize extraordinary measures to fight the pandemic (Nunes 2020; Wiessmann 2020).

A review of COVID-19 research suggests a distinction between different types of existential threats, such as to businesses (Donthu and Gustafsson 2020; Enriques 2020), including the risk of bankruptcy (Wang et al. 2020), or to individuals, where feelings of existential anxiety and threat were mentioned (Peteet 2020), for instance due to loss of employment or to the failure of small businesses (Blustein and Guarino 2020), or severe health impacts including death (Fuchs 2020). Furthermore, COVID-19 has repeatedly been discussed in relation to the climate crisis, e.g., in terms of the need to mitigate ecological degradation and strengthen conservation to prevent transmission of viruses from wildlife animals (Baiker et al. 2020; Carlson et al. 2022), or in terms of how compound risk and crises can be managed (Phillips et al. 2020; Salas et al. 2020; Ebi et al. 2021a).

The COVID-19 pandemic is extraordinary because it simultaneously affected well-being and health of a large part of the world’s population, in different ways and to different extents, such as by limiting healthcare and by causing infection; health effects and, for some, death; emotional distress and mental illness; domestic isolation and domestic violence; lack of movement and social contact; loss of jobs and income and economic problems; and bottlenecks in supply of food and goods (Evans et al. 2020; Döring 2020). With relevance for climate change risks, we recognize the context- and site-specific conditions that shaped vulnerabilities and exposure to COVID-19, the differentiated behavior and response on an individual, societal, or national level, and the interconnectedness of socio-economic systems.

### Synthesis

Throughout this section, we have seen a range of scientific contexts and scopes in which existential risks have been framed and defined. In the GCR community, existential risks are of global scale, with gradual or abrupt loss of potential, collapse, or even extinction of humanity. The civil society climate movement takes on these imaginaries of collapse but often more in a sense of the need of fundamental change. Key risks and RFCs in the IPCC assessments do not escalate to the risk level of GCRs but encompass a range of spatial scales from local to global, and are more nuanced in terms of climate-related threats to human health, land and property, income, livelihoods or identity, and culture. The COVID-19 pandemic adds a tangible aspect of lived reality and shows how a threat that has repeatedly been termed “existential” can affect a range of populations, from individuals to nation states where the large majority was “only” affected in terms of well-being. With this background, we now develop a definition of existential risks in the scientific context of climate change.

## Elements and dimensions of the existential risk space of climate change

### Levels of severity relevant for existential threats

**Fig 1:**
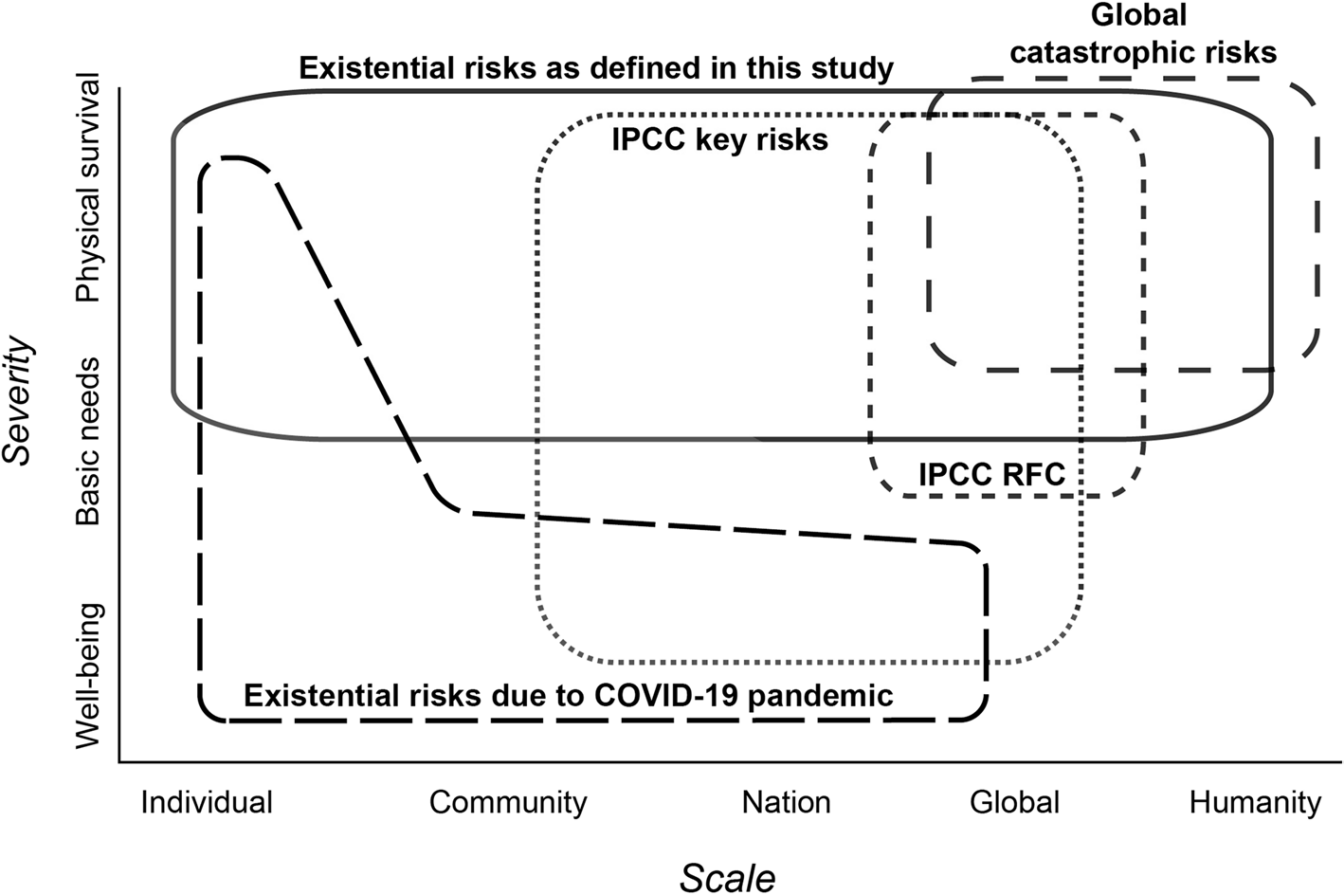
Different narratives and concepts relevant or referring to existential risks, identified between an axis of scale from individual to global and humanity and an axis of the severity of the way how the subjects are affected, distinguishing three levels from well-being to physical survival. For the IPCC, we distinguish between key risks and Reasons for Concern (RFC), with the first ones extending down to a local level, while RFCs have a global focus. Existential risks as defined in this study cover a broad range of scale from individual to humanity, while the severity range is limited to physical survival and basic needs

In view of the lack of a definition of existential risks in the climate change context, we propose the following definition: existential risks are those that threaten the existence of a subject, where this subject can be an individual person, an entire community, a nation state, or humankind. This definition hence covers a range of scales of people being affected, from local to global (Fig. 1). To further specify the threat of existential risks, we propose three different levels of severity: (1) physical threat to human life; (2) threat to basic human needs, such as water, food, health, and shelter; and (3) conditions or risks that undermine the pillars of living standards for acceptable levels of well-being. We acknowledge that what counts as “basic” and “acceptable” differs across cultural, social, and political contexts.

These three levels of severity determine how the existence of a subject is put at risk. Threats to the physical life represent the most existential level; threats to basic needs can also be existential in the sense of our definition. Level 3, i.e., threatened well-being, however, would typically not fall within existential risks but there may be exceptions (e.g., when a nation or community is unable to provide acceptable levels of well-being to their population, the nation’s or community’s survival may be undermined).

To what extent a subject is affected by an existential risk depends on the hazard, and its exposure and vulnerability, and can vary among and between individuals, communities, countries, and regions. For instance, whether COVID-19 is a risk to life depends mainly on the vulnerability and, to some extent on the exposure, of the affected person. For people with critical health conditions or the elderly, COVID-19 may be a risk to their life, while for most others, it may affect their well-being and undermine some of the basic elements of acceptable living conditions, e.g., by loss of job and economic income.

The definition of acceptable levels of well-being and living conditions is a long-standing discussion in development research and practice, as well as in philosophy and ethics (Sen 1979; Maslow 1987; Daniels 1990), and essentially is beyond the scope of this contribution. The basic needs approach, initially developed in the 1970s, seeks to define minimum resources for and standards of physical well-being, as a measure of poverty (Jolly 1976), often including food, water, shelter, and clothing, and in some cases also incorporated sanitation, education, and healthcare (Denton 1990; Sarlo 1992). The concept has been criticized as too narrow (Schuppert 2011; Siebel and Schramme 2020) and overly focused on consumption, resulting in the emergence of the concept of capabilities as an alternative (Sen 1992; Sen and Nussbaum 1993; Nussbaum 2000). Capabilities are defined either similar to basic needs, or they can stand for a level of well-being of adequate living conditions, while some researchers have emphasized that the concept established thresholds which are connected to specific societal contexts, i.e., the aforementioned elements remain the same but quantity and quality are relative to the respective society (Sarlo 2013).

The concepts of life-threatening conditions, basic needs, and capabilities are reflected in discussions about habitability that are gaining traction in the climate change debate (Wallace-Wells 2019). The IPCC defines habitability as “the ability of a place to support human life by providing protection from hazards which challenge human survival, and by assuring adequate space, food and freshwater” (IPCC 2019a, b, p. 688). The consideration of protection from hazards which challenge human survival alludes to life-threatening aspects, while the assurance of space, food, and freshwater refers more to the notion of basic needs. Building on these ideas, Duvat et al. (2021) use a concept of habitability pillars for atoll islands, in which they include a component of access to sustainable economic activities. However, the most basic existential aspect of habitability is that of survival at a specific location, threatened, e.g., by sea-level rise, floods, and storm surges or excess heat. Indeed, there are already locations where sea-level rise and coastal erosion lead to decisions to retreat, demonstrating the existential dimension for its inhabitants, be it involuntary (Buser 2020), or voluntary through buy-out programs (Siders et al. 2019; Mach and Siders 2021).

Excess heat is often seen as the most direct limit to human survival in the context of existential risks and climate change (Ord 2020). The wet-bulb temperature (*T*_W_) is commonly used as a metric of meteorological and associated human physiological conditions that indicate survivability. Although somewhat debated, a *T*_W_ of 35°C is taken as the upper limit in which survival under sustained exposure is even under idealized conditions no longer possible (Sherwood and Huber 2010; Raymond et al. 2020b; Pörtner 2021). While observational evidence suggests that a *T*_W_ of 35°C has so far only been exceeded in very few exceptional cases, heat mortality has been observed to occur at *T*_W_ levels far below 35°C, e.g., at *T*_W_<28°C during the 2003 European heat wave or the 2010 Russian heat wave. Weather services therefore operate with levels of “dangerous” heat, which, for instance, are indicated using a heat index (HI, reflecting temperature and humidity effects) with a threshold of HI = 40.6°C in the USA (Matthews et al. 2017). Dangerous heat levels could affect well-being and basic needs, as well as represent life-threatening conditions, depending on individuals’ vulnerability and exposure. The *T*_W_ limit is unambiguously life threatening in an ultimate form. Studies project that dangerous heat stress conditions, if unabated, could affect hundreds of millions of people under warming of 1.5 and 2°C above preindustrial levels during this century, especially when combined with urban heat island effects (Matthews et al. 2017; Marcotullio et al. 2021). Medium to high emission scenarios would bring hotspot regions, such as South and Southwest Asia, beyond the limit of survivability (Pal and Eltahir 2016), and extreme high-end scenarios (beyond 7°C or even 10°C) would render large parts of the world uninhabitable (Sherwood and Huber 2010).

Basic needs and well-being are threatened in multiple ways by climate change. The IPCC AR6 assessed a range of impacts of climate change that affect basic needs and well-being, including increasing water scarcity limiting access to water, food production, infectious diseases affecting health, or loss of identity, place attachment, and social community cohesion by migration or relocation (IPCC 2022). Climate change–related risks can penetrate through all three levels of severity relevant for existential risks as detailed above; for instance, floods can be life-threatening, they can affect basic needs by damaging houses (shelter) and destroying water and sanitation services, and they can affect various aspects of well-being related to mental conditions (e.g., by disaster-related traumas), or to disruption of daily life practices.

### Dimensions of an existential threat

In the previous section, we have proposed a definition of existential risk and have outlined three levels of severity where the first two levels clearly fall into the existential risk space and the last one only exceptionally. Our definition is different from those that are proposed in the GCR community (Torres 2019) in that it comprises a range of scales from individuals to nations. Our definition is intended to position existential risks in the context of climate change, and hence the question arises how they relate to established levels of risks in the climate change community, in particular in the IPCC. In the IPCC, RFCs refer to risks that may be of global concern, while representative key risks may be of concern only locally or for certain population groups (Fig. 1) (O’Neill et al. 2022). Furthermore, O’Neill et al. (2017, 2022) define the transition from moderate to high and very high risk levels as a point where impacts become severe, widespread, and irreversible along criteria defined for key risks (cf. Section 2.2). The term “widespread” for high or very high risk levels is not precisely defined in the IPCC, but generally alludes to larger spatial scales. Our definition of existential risks builds on the IPCC risk framing but is not simply the next level of escalation following from the very high risk level of the IPCC. Rather, it also extends to the scale of individuals but the very high severity (i.e., existential) levels of risks persist across the different scales (Fig. 1).

To make existential risks more useful, specific, and tangible for climate change risk research, we therefore suggest specifying them along additional dimensions. We list here six dimensions which build on (WBGU 1998; Oppenheimer et al. 2014; O’Neill et al. 2017, 2022; Renn et al. 2019) (1) the processes and mechanisms of threat, (2) systems affected by existential risk, (3) the magnitude of threat, (4) the probability of occurrence of the threat, (5) the time horizon, timing, and speed of the process, and (6) the scale. Our objective here is to define the characteristics of the dimensions that frame and define existential risk, where the threshold between non-existential and existential risks is given by our definition.

1. *The processes and mechanisms of the threat*: We think that it is essential to clearly identify and trace the physical and social processes through which an existential threat is functioning. In the context of climate change, risks have their origin in climate- and weather-related processes and then propagate through biophysical and social systems (Huss et al. 2017; Huggel et al. 2019). Both sudden and slow-onset processes may represent the origin of existential threats and can include, for instance, storms and floods and landslides, extreme heat and drought, coastal erosion, and loss of snow and glacier ice. Tracing processes from source to affected people and systems will help identifying the existential threat, and specifically, support analyzing how and to what extent the first and second severity levels that define existential risks (i.e., threat to human life, basic needs, respectively) can be affected. It may be useful to distinguish between impacts that directly and indirectly affect people; for instance, heat and floods have a direct (physical) impact on people but typically also produce indirect impacts when propagating through interconnected systems (Simpson et al. 2021).
2. *Systems affected by existential threats*: We define existential risks as a threat to the existence of a person or a group of people, mediated through processes (as outlined above) that affect people’s lives and basic needs. These, in turn, are affected by climate change–related impacts on specific systems, for instance, on food production and supply, on water resources and availability, on infrastructure, energy supply or ecological systems, and on habitable and arable land. Because these systems are interconnected, a threat typically affects more than one system or object type (Reichstein et al. 2021). For example, a major flood disaster may directly threaten people’s lives but also affect infrastructure and health services, which in turn can threaten the lives or basic needs of additional people when health services collapse. Effects of climate change on intangible values may often affect well-being and thus not qualify for existential risks in our framing. However, there are crucial exceptions; for instance, loss of glaciers and snow in mountains affects cultural identity in the Andes and Himalayas, and Indigenous people in the Andes have reported that their community would no longer exist if glaciers are gone (Diemberger et al. 2015; Jurt et al. 2015).
3. *The magnitude of the threat*: Related to the previous dimension, we seek to identify the magnitude of the threat and its consequences. In some cases, this dimension may be expressed by simple and well-known metrics, such as the height and area of flooded inhabited land, temperature, and drought indices or the amount of available water. Some of these metrics are directly relevant for existential risks; for instance, a *T*_W_ > 35° represents a threat to physical life, or an amount of available water lower than some defined standard affects basic needs. These metrics, hence, define existential risks, because they indicate certain thresholds beyond which risks become existential, according to scientific or societal standards. Recent examples where extraordinary high magnitudes were reached are the devastating 2021 floods in western Europe with statistically extremely high rainfall intensities and flood discharges (Fekete and Sandholz 2021), or the heatwave (heatdome) that struck the northwestern USA and southwestern Canada in 2021 with extremely high temperatures close to 50°C (Henderson et al. 2022). Other types of metrics for magnitude relate to the number of affected people, the geographic or administrative area affected (e.g., from provincial to national scale), the area of arable land, or the number of systems or sectors affected (e.g., food and energy production systems) (IPCC 2014, 2018, 2019b; Hirabayashi et al. 2021). These metrics may also be relevant to define existential risks, but scale is here important, i.e., a climate-related risk that threatens the existence of a community may not be existential for the nation state of the community (and maybe not for all individuals of the community). This is why we emphasize the importance of indicating the precise scale of existential risks (cf. dimension 6 below).
4. *Probability of occurrence of the threat*: Although probability of occurrence is a central component of hazard and risk analysis, it is often not explicit or specified in the discussion about existential risks. This may be related to the difficulties of estimating the probability of occurrence of specific types of events. Ord (2020) made an attempt to estimate the probability of occurrence within the next 100 years for several existential risks that are of a dimension that may permanently destroy humanity’s potential for desirable future development and thus severely limit the futures that remain open to humanity. He found a probability of 1:1,000,000 for an asteroid impact, 1:10,000 for a super-volcano eruption, 1:30 for an engineered pandemic, and 1:1,000 for catastrophic climate change. This is one of the rare quantitative estimates of the probability of catastrophic climate change, given by scenarios of warming in excess of about 10°C (above preindustrial levels), including a scenario with a runaway greenhouse gas effect (Ord 2020). This effort differs greatly from the typical concerns of climate science in terms of probability of occurrence of weather and climate phenomena. Climate change research has a long record in calculating probabilities of occurrence for different types of extreme weather events, floods, or landslides (IPCC 2012, 2018). Indication of probability of occurrence for slow-onset processes that would lead to existential risks, such as extreme sea-level rise, is much more difficult. In some cases, it may not be feasible because the probability of occurrence of many slow-onset processes is high, close, or equal to one, and the focus (and also the uncertainty) is more on the speed and magnitude of the arrival and evolution of the event (Mechler et al. 2019).
  Hence, the estimates of the probability of occurrence for risks that have existential character need to specify the natural and social processes involved, starting from the trigger events (e.g., a heatwave or a storm), analyzing the possible impact cascade, and considering the exposure and vulnerability of the location (or region) that may combine to result in existential risks for a fraction of the population. Even though quantitative figures may only be possible for the trigger events, a qualitative estimate will be useful for the purpose of risk management response.
5. *Time horizon, timing, and speed of process*: Some types of existential risks, such as asteroid impacts or a nuclear war, strike immediately with devastating global consequences over the following months or years. In a climate change context on a global scale, there is no analogue to this rapidity, as the time scales are longer (e.g., collapse of the West Antarctic Ice Sheet), or consequences are spatially more constrained (e.g., tropical cyclones). If climate change is framed as a threat to human civilization, then this threat is not immediate but would unfold over decades and centuries, as for instance with sea-level rise (see Tol et al. 2006). There is sufficient historical evidence that local or regional societies typically “collapse” (or decline) over extended periods of time (decades to centuries) (Diamond 2005; Butzer and Endfield 2012), even though ancient societies may not be an adequate reference for modern society given the much higher levels of technology and other capacities today. A globalized world and highly interconnected systems may actually increase the speed of risk propagation across countries and continents. Timing can also be decisive in terms of repetitive events, as populations can recover from being struck by one flood, but if flood events become repetitive or severe, populations may decide to retreat (Nelson 2014; Siders et al. 2019; Haasnoot et al. 2021), as the risk becomes existential, and adaptation limits are reached (Gharbaoui and Blocher 2018; Nalau and Handmer 2018; Mechler et al. 2020). Adaptation limits, defined by the limit when technical and societal options to eliminate or reduce such risks are not available anymore (Wallimann-Helmer et al. 2021; O’Neill et al. 2022), are highly relevant because the scale, severity, and possibly speed of the changes that would lead to existential changes may overwhelm the technical, social, and economic options to adapt. This has been shown in policy exercises for rapid and extreme sea-level rise of up to 5 m, where it is possible that technical capacities to adapt may exist, but societal and institutional processes lead to limits and eventual abandonment of coastal zones (Tol et al. 2006; Olsthoorn et al. 2008). The timing and speed are also relevant when different hazards and risks combine, are compounded, and produce cascading impacts (Raymond et al. 2020a; Reichstein et al. 2021).
6. *Scale*: Scale of what is affected, from individual to humanity, or from local to global, may be the most fundamental dimension in the discussion about existential risks, and represents a main source of ambiguity in the debate. For GCR, the scale is necessarily global, and any local or regional risk would not count towards this sort of high-end risk (Ord 2020). In the recent past, the term “existential” has often been used in media or public discourse for events that affected large geographic areas. Examples of such climate risk events include, for instance, the 2017 coastal El Niño affecting much of the South American west coast (Rodríguez-Morata et al. 2019), the heat and drought affecting central Europe in 2015 and 2018 (Ionita et al. 2017; Toreti et al. 2019), the 2019/2020 Australian bushfires (van Oldenborgh et al. 2021), or the abovementioned 2021 floods in western Europe and the 2021 heatwave in the northwestern USA and adjacent portions of Canada.

In the sense of our definition, these events were existential for a substantial number of people and some smaller communities (in the case of the floods in Germany) but not for the larger societies in these regions. Our definition of existential risks, in fact, is not bound to a specific scale but encompasses all scales from individuals to humanity. This implies that if we talk about existential risks, we need to specify the scale of affected people or social/administrative structure.

It is also important to underline that in our framing, the scale is not a criterion to define existential risks; however, it may be used to indicate the level of existential risks. For instance, an existential risk to a nation state may be considered a high level of existential risk, while an existential risk to some individuals only a low level of existential risk. However, here, we do not introduce or further elaborate on different levels of existential risks. Rather, we illustrate the case with Fig. 1, where we sketch the scale from individual to humanity versus the different levels of severity as described in Section 3.1, for different narratives and types of risks. Our definition positions existential risks in the high severity space but it covers a range of threatened people and societies that needs to be defined for each analysis. In contrast, GCRs only refer to the largest scale of affected people, i.e., humanity. The IPCC, as outlined above, uses the criteria of “widespread,” “significant,” and “severe” for high and very high risk levels (for key risks) (O’Neill et al. 2022). Based on this and on how key risks and RFCs are assessed in the IPCC AR6, we locate key risks in a range from multiple communities to nation and global and RFCs only in the global scale (Fig. 1). On the severity scale, we position IPCC risks somewhat below existential risks as defined in this paper, but are considerate that for RFCs limited specification with respect to the severity level is indicated in IPCC reports. In fact, the exact position of IPCC risks in this matrix is an open question and our contribution can hopefully foster discussions such as for the next IPCC cycle. The existential risk space perceived by the climate movement is difficult to locate because of lacking precision in the discourse and therefore we did not include it in Fig. 1.

## Conclusions and perspectives

In this contribution, we first draw upon various concepts, narratives, and research fields concerned with existential risks, with which we set the context of the debate on existential risks of climate change. Research, particularly at the science-policy interface in boundary organizations, such as the IPCC, has pointed to the existential issues that are increasingly observed, e.g., in retreat and relocation in coastal and mountain regions.

We offer the following contributions to the scientific community: firstly, a clear definition of existential risks which is based on two levels of severity, i.e., threats to the physical life and to basic human needs. The definition encompasses a range of scales of affected subjects, from individuals to communities, nation states, and humanity. Implicit in our definition is, secondly, the need to specify key dimensions of existential risks of climate change. Key among these dimensions are the two axes displayed in Fig. 1, i.e., the level of severity (from well-being to basic needs and life threatening) and the scale of affected subjects, from individuals to humanity. We develop a set of additional dimensions such as physical and social processes involved, systems affected, magnitude, scale, and timing and probability of occurrence that facilitate a more nuanced and hence useful analysis of existential risks.

Moreover, making the existential space part of risk analysis or risk management strategies can help to identify which risks may need to be avoided, either through adaptation and risk reduction or through climate change mitigation, or, in the worst case, define conditions where risks may not be further reduced, and transformative change becomes necessary. The need for such measures that deeply affect social, economic, or governance systems also shows the relevance of existential risks for questions of justice where a more precise definition of this type of threat has implications for the definition of entitlement of affected individuals, communities, or nation states.

In conclusion, our intention with this paper is to offer a framework, context, and definition of existential risks of climate change to promote and facilitate further discussion and research on the topic. Moreover, we suggest to more explicitly consider existential risks in risk assessments, and eventually in risk management strategies and actions.

## Data Availability

Not applicable

## Footnotes

1 We use two terms, threat and risk, to refer to negative outcomes which result from the interactions between hazards and socio-economic conditions. We use risk as a technical term, defined as the probability of a negative outcome, or more broadly, as defined in recent IPCC reports, the potential for adverse consequences (IPCC 2018). Threat is used here as a less technical and less specific term, referring to a condition in the future that may cause harm, without being explicit about a probability of occurrence and the level of negative outcome.
